# Extramedullary hematopoiesis in sickle cell anemia

**DOI:** 10.36416/1806-3756/e20250516

**Published:** 2026-08-13

**Authors:** Alessandro Severo Alves de Melo, Glaucia Zanetti, Edson Marchiori

**Affiliations:** 1. Universidade Federal Fluminense, Niterói (RJ) Brasil.; 2. Universidade Federal do Rio de Janeiro, Rio de Janeiro (RJ) Brasil.

**Figure 1:**
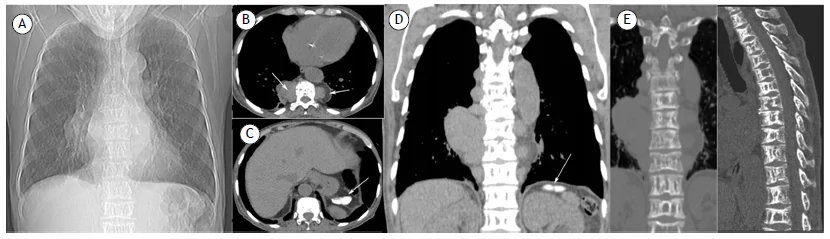
In A, posteroanterior chest radiograph showing bilateral paravertebral masses. In B-D, chest CT images confirming the presence of multilobulated, heterogeneous masses with soft-tissue density and areas of fat inside (-45 HU; arrows in B). Note also the significant reduction in the volume of the spleen, which appears calcified (arrows in C and D). In E and F, anteroposterior and lateral radiographs of the thoracic spine showing changes in the vertebral body structure consistent with sickle cell anemia.

A 60-year-old man was hospitalized with persistent cough and fever of recent onset, which progressed to a decline in his general condition. He had sickle cell anemia that had been treated irregularly, in association with congestive heart failure, liver cirrhosis, and perimalleolar ulcers. On physical examination, he was alert and oriented, pale, and emaciated. Laboratory tests showed normocytic/normochromic anemia. A chest CT examination showed changes consistent with extramedullary hematopoiesis (EMH; Figure 1). The patient underwent antibiotic therapy and blood transfusion; he showed clinical improvement and was discharged from the hospital.

EMH is a compensatory physiological response to chronic hypoxia due to anemia, secondary to inadequate bone marrow function or ineffective hematopoiesis, characterized by the formation of normal blood cells outside of the bone marrow. It is usually seen in patients with hematological conditions, including hemoglobinopathies (e.g., thalassemia and sickle cell anemia) and myeloproliferative disorders.^1^ ^-^ ^3^ The diagnosis of intrathoracic EMH can be safely made when radiological findings are present together with chronic anemia. Radiographically, intrathoracic EMH usually presents as multiple paravertebral masses with soft-tissue and/or fatty densities, usually bilateral and lobulated with well-defined borders. Biopsy is often avoided, as it carries the risk of catastrophic hemorrhage.^1^ ^-^ ^3^ In conclusion, the finding of well-defined, bilateral paravertebral masses in patients with previously diagnosed blood disease should alert physicians to the diagnostic possibility of EMH.
